# Effects of modified uvulopalatopharyngoplasty combined with pharyngeal and genioglossus exercises on sleep quality, cognitive function, and quality of life in patients with obstructive sleep apnea-hypopnea syndrome: a retrospective study

**DOI:** 10.3389/fsurg.2026.1747801

**Published:** 2026-06-01

**Authors:** Pingping Zhang, Dawei Liang

**Affiliations:** Department of Otorhinolaryngology, The First People’s Hospital of Linping District, Hangzhou, China

**Keywords:** cognitive function, modified uvulopalatopharyngoplasty, obstructive sleep apnea-hypopnea syndrome, pharyngeal and genioglossus exercises, quality of life, sleep quality

## Abstract

**Objective:**

To evaluate the effects of modified uvulopalatopharyngoplasty (UPPP) combined with pharyngeal and genioglossus exercises on sleep quality in patients with obstructive sleep apnea-hypopnea syndrome (OSAHS).

**Methods:**

A total of 99 OSAHS patients who underwent modified UPPP were divided into a control group (*n* = 54, conventional postoperative training) and a study group (*n* = 45, pharyngeal and genioglossus exercises in addition to conventional postoperative training). The evaluation indicators included upper airway remodeling, respiratory pressure, sleep quality, sleep architecture, cognitive function, quality of life, and psychological state. Sleep quality was assessed using the Stanford Sleepiness Scale (SSS) and Consensus Sleep Diary (CSD).

**Results:**

At 6 months postoperatively, the study group demonstrated better outcomes compared to the control group in terms of upper airway remodeling, respiratory pressure, sleep quality, sleep architecture, cognitive function, and quality of life (*P* < 0.05). The SSS score in the study group was significantly lower than that in the control group. Postoperative self-rated sleep quality in the study group was superior to that in the control group (*P* < 0.05), with a greater proportion of patients in the study group rating their sleep quality as “good” or “very good” compared to the control group.

**Conclusion:**

The combination of modified UPPP and pharyngeal and genioglossus exercises was associated with improvements in sleep quality, cognitive function, and quality of life in patients with OSAHS, and resulted in better clinical outcomes compared with conventional training.

## Introduction

Obstructive sleep apnea-hypopnea syndrome (OSAHS) is a common sleep-related respiratory disorder characterized by hypoventilation, apneas, and hypoxemia resulting from upper airway obstruction during sleep. This condition profoundly affects sleep quality and may result in multi-systemic damage (1, 2). The incidence of OSAHS is estimated at 5%–15%, with the risk increasing significantly with age. In untreated individuals, the 5-year mortality rate may reach 11%–13% (3). Current clinical management primarily involves surgical and conservative interventions. Modified uvulopalatopharyngoplasty (UPPP), as one of the classic surgical approaches, alleviates intermittent velopharyngeal airway obstruction and enhances upper airway patency. However, studies suggest that the efficacy of this procedure is approximately 41%, with a potential risk of postoperative upper airway collapse, which may limit its overall efficacy (4).

The collapse of the upper airway is strong associated with the onset of OSAHS, primarily due to neuromuscular dysfunction and an increased anatomical load on the upper airway. The genioglossus and velopharyngeal muscles, essential for maintaining upper airway patency (5), often exhibit functional decline in patients with OSAHS. Therefore, postoperative combined exercises targeting both the pharyngeal and genioglossus muscles may improve the function of upper airway muscles, alleviate airway collapse, and enhance surgical outcomes (6).

The aim of this study was to investigate the effects of modified UPPP combined with pharyngeal and genioglossus exercises on sleep quality, cognitive function, and quality of life in patients with OSAHS. It was hypothesized that the combined intervention may help improve sleep quality in patients with OSAHS.

## Materials and methods

### Clinical data

Clinical data from 99 patients with OSAHS admitted to our hospital between March 2018 and February 2022 were retrospectively analyzed. The study population comprised 56 male and 43 female patients, aged 28–68 years (mean: 52.1 ± 6.2 years), with a body mass index (BMI) ranging from 18.5 to 29.4 kg/m^2^ (mean: 25.54 ± 1.97 kg/m^2^) and a disease duration of 3–18 years (mean: 11.85 ± 2.35 years) (disease duration was determined based on the patient's medical records, calculated from the date of the initial OSAHS diagnosis to the date of admission evaluation, as verified by a specialist according to the medical history). The severity of OSAHS was classified as mild (16 patients), moderate (49 patients), and severe (34 patients). All patients underwent modified UPPP. In this retrospective study, patients were grouped according to the postoperative rehabilitation training they received. Those who received conventional training were included in the control group (*n* = 54), whereas those who received additional pharyngeal and genioglossus exercises on the basis of conventional training were assigned to the study group (*n* = 45). This study was approved by the Ethics Committee of The First People's Hospital of Linping District. All procedures involving human participants were performed in accordance with the ethical standards of the institutional and/or national research committee and with the 1964 Helsinki Declaration and its later amendments or comparable ethical standards. Informed consent was obtained from all participants.

**Figure 1:**
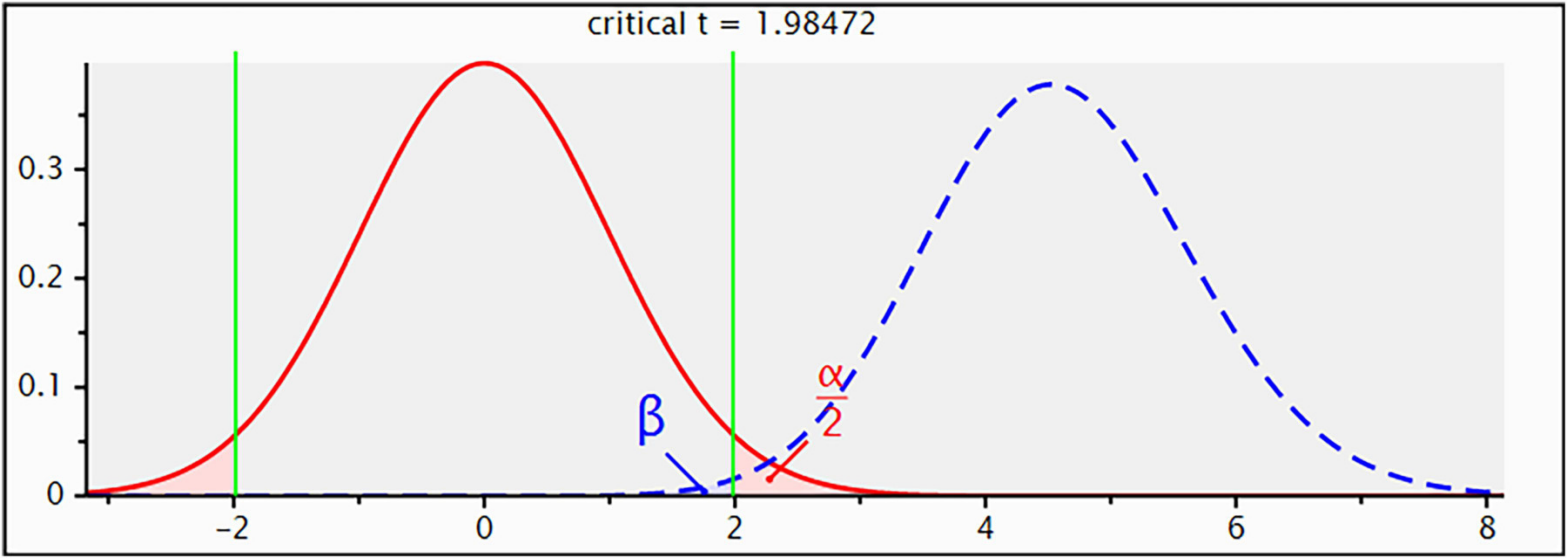
*Post hoc* power analysis.

This study is a retrospective analysis. Although *a priori* power analysis was not conducted during the study design phase, a *post hoc* power analysis was performed to validate the adequacy of the sample size. Based on previous literature (7), the SSS score at 6 months postoperatively was selected as the primary endpoint to assess sleep quality. The effect size between the study and control groups was calculated as Cohen's *d* = 0.92. The sample sizes of the control group (*n* = 54) and the study group (*n* = 45) were analyzed using G*Power software (8) at the significance level of *α* = 0.05, yielding a statistical power of 0.9947 (Figure 1). These findings indicated that the sample size was adequately powered to detect significant intergroup differences with a probability of 99.47%.

### Inclusion criteria

(1) Diagnostic and grading criteria. OSAHS was diagnosed according to the criteria outlined in the Guideline for Primary Care of Adult Obstructive Sleep Apnea (2018) (9), and the severity was classified based on the Apnea Hypopnea Index (AHI) as follows: mild, with a nocturnal lowest blood oxygen saturation (LSaO2) of 85%–90% and an AHI of 5–15 events per hour; moderate, with an LSaO2 of 80%–85% and an AHI of 15–30 events per hour; and severe, with an LSaO2 of ≤80% and an AHI of >30 events per hour.
(2) Inclusion criteria. Patients who met the above diagnostic criteria for OSAHS, including those with mild, moderate, or severe OSAHS, aged 18–80 years, and with complete clinical data available for analysis, were included in the study.
(3) Exclusion criteria. Patients with the following conditions were excluded: concurrent infectious diseases such as viral hepatitis, syphilis, and AIDS; recent use of oxygen-enhancing drugs or sleep medications; blood disorders such as leukemia and aplastic anemia; respiratory diseases such as pulmonary infections and bronchiectasis; and mental illnesses, intellectual disabilities, or speech disorders. The use of Drug-Induced Sleep Endoscopy (DISE) to identify the site of obstruction facilitated the assessment of both the anatomical and functional status of the upper airway (10). Additionally, CT scans were employed to assist in evaluating the airway's anatomical structure, excluding potential anatomical abnormalities and ruling out patients with obstruction located beyond the palatopharyngeal plane. Patients with maxillofacial deformities and sleep disorders such as narcolepsy and central respiratory arrest were also excluded.

## Methods

### Modified UPPP

**Figure 2:**
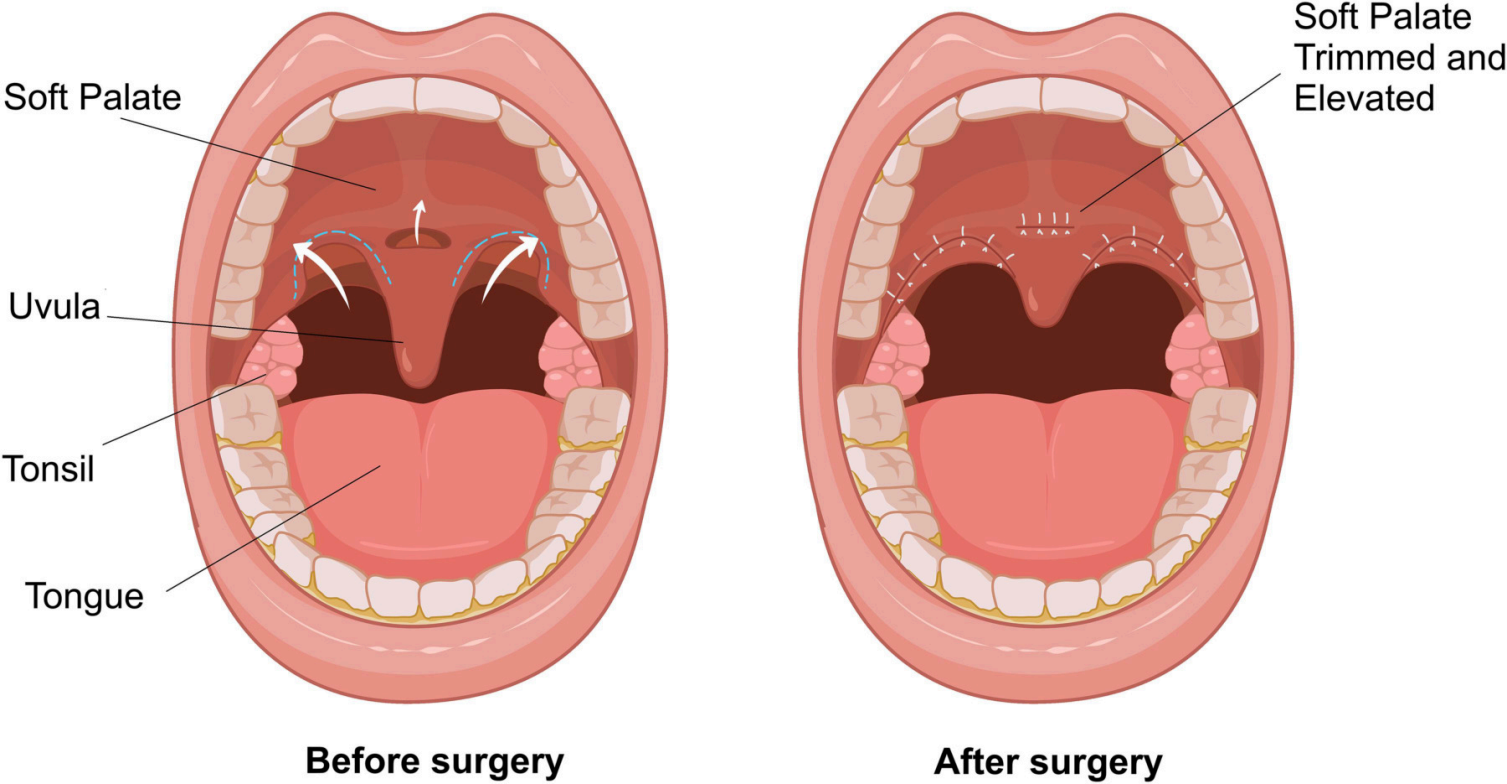
Illustrating figure of modified UPPP.

In this study, all surgeries were performed by a team of experienced surgeons to ensure consistency in surgical techniques and expertise. The surgical protocol and postoperative management adhered to standardized procedures, including preoperative evaluation, individualized surgical plans, uniform postoperative care, and consistent follow-up schedules. These measures minimized potential biases related to variations in surgical practice and medical protocols, ensuring comparability between the two groups of patients. Both groups underwent modified UPPP. During the implementation of the modified UPPP, general anesthesia was administered via nasal intubation, ensuring proper management of the patient's airway. The surgeon carefully exposed the palatine velum gap, bilateral tonsils, the palatoglossal arch, and the palatopharyngeal arch, making individualized adjustments based on the patient's anatomical structure (Figure 2). Initially, intraoperative assessment of the tonsils’ size and their impact on airway patency was performed. In patients with grade II or higher tonsillar hypertrophy, concurrent tonsillectomy was conducted to reduce pharyngeal obstruction, enhance airway patency, and minimize the risk of postoperative retroglossal obstruction. During the excision, great care was taken to preserve adjacent tissues, avoiding excessive trauma to reduce postoperative bleeding and discomfort. In cases where the tonsils were relatively small or showed no significant hypertrophy, preservation was preferred to avoid unnecessary tissue damage. After completing the tissue cleaning, the adipose tissue within the palatine velum gap was meticulously excised, while the excess tissue in the palatoglossal and palatopharyngeal arches was moderately trimmed to optimize the airway structure. Subsequently, the surgeon precisely suspended the superior or posterior portion of the palatopharyngeus muscle, securing it to anatomical positions that optimally supported the upper airway, such as the posterior margin of the hard palate, the lateral pharyngeal wall, or the pharyngeal apex. Tension-free suturing techniques were used to ensure the stable recovery of the muscular tissue. During the suturing, meticulous attention was paid to the alignment of the mucosa, minimizing the risk of postoperative scarring. Furthermore, in cases of hypertrophic or excessively elongated uvula, a moderate truncation was performed during surgery to restore it to a reasonable shape, thereby preventing airway obstruction during respiration. To further optimize pharyngeal ventilation, the surgeon expanded both the transverse and anteroposterior dimensions of the pharyngeal cavity, reducing the occurrence of apnea or hypoventilation caused by airway constriction. This procedure, through the precise excision of excess tissue, optimization of velopharyngeal muscle function, and expansion of the pharyngeal cavity, aimed to effectively alleviate upper airway obstruction in patients with OSAHS, thereby enhancing sleep quality, cognitive function, and overall quality of life.

### Postoperative training

Previous literature (11) and clinical practice have demonstrated that long-term management through orofacial myofunctional therapy (OMT) combined with respiratory training can enhance upper airway muscle function, improve breathing patterns, and thereby mitigate the severity of OSAHS. Consequently, it is strongly recommended in clinical treatment that patients undergo targeted exercises to improve therapeutic outcomes.

The control group underwent conventional postoperative training, which consisted of a series of standardized breathing and rehabilitation exercises aimed at restoring respiratory function, improving airway condition, and enhancing postoperative recovery. Conventional training included lip contraction and diaphragmatic breathing exercises. For lip contraction training (12), patients were instructed to keep their mouth closed, inhale through the nose, hold their breath briefly, then open their lips and gently contract them while exhaling slowly through the mouth, with the lips shaped like a whistle. The ratio of exhalation time to inhalation time was 2:1 or 3:1. This exercise was performed for 5 min per session, three times daily. For diaphragmatic breathing exercises (13), patients were positioned supine with both hands placed on the upper abdomen. During exhalation, the abdomen was contracted, and gentle pressure was applied with both hands towards the abdomen. During inhalation, the abdomen was distended, with both hands expanded outward. Patients performed this exercise three times daily for approximately 5 min per session, gradually transitioning to a natural diaphragmatic breathing pattern.

In addition to conventional training, patients in the study group also performed the following exercises (14): mouth opening and tongue stretching (10 repetitions, twice daily); massage of the submental region using the thumb pulp (1 min), followed by upward and forward push of the submental region with the thumb (10 repetitions, 5 s each); cheek puffing (10 repetitions, 5 s each); forced expiratory effort with nasal occlusion and a closed mouth (10 repetitions, 5 s each); and deep nasal breathing with the mouth closed (20 repetitions). Both groups underwent continuous training for 6 months. During the follow-up period, medical staff reminded patients to adhere to the training through regular follow-up, thereby improving training adherence.

### Observational indicators

#### Upper airway remodeling and respiratory pressure

Upper airway computed tomography (CT) was performed using a Siemens SOMATOM sensation 64-slice CT system. The anteroposterior, transverse, and cross-sectional areas of the narrowest part of the palatopharynx were assessed preoperatively and 6 months postoperatively. Maximal inspiratory pressure (MIP) and maximal expiratory pressure (MEP) were measured using a pulmonary function apparatus (Model: X1, XEEK (Xiamen) Medical Equipment Co., Ltd.).

#### Evaluation of sleep quality

The sleep quality of patients was assessed preoperatively and 6 months postoperatively using the Stanford Sleepiness Scale (SSS) and the Consensus Sleep Diary (CSD). (1) The SSS (15) is a subjective measure of individual sleepiness, developed by Hoddes et al. in 1973. This simple and rapid self-assessment tool is primarily employed to measure an individual's level of wakefulness or drowsiness at various time points. This scale categorizes subjective sleepiness into seven levels, ranging from 1 to 7 in increasing severity. A score of 1 signifies a state of full alertness, vigilance, and vitality; scores of 2–3 indicate normal functioning or mild drowsiness with preserved focus; scores of 4–5 denote significant sleepiness, impaired attention, and difficulty maintaining wakefulness; while scores of 6–7 reflect extreme drowsiness, marked inattentiveness, and imminent sleep propensity. This scale was used to assess sleep quality and daytime sleepiness. (2) The CSD scale (16), developed by Carney et al. in 2012, is used to standardize self-rated sleep diary assessments. Formulated through a comprehensive analysis of various existing sleep diaries, it aims to provide a consistent and reliable method for sleep data collection, facilitating its broad application in both clinical and research settings. It consists of nine items: time of going to bed, time attempting to fall asleep, duration of time taken to fall asleep (from the attempt to sleep to actual sleep), number of awakenings, total wake time, time of waking in the morning, time of getting out of bed, self-rated sleep quality (with five levels: very poor, poor, fair, good, very good), and additional comments from the participants. All tasks must be completed within one hour of waking up in the morning. The evaluation of sleep quality was based on three key indicators: duration of time taken to fall asleep, number of awakenings during the night, and self- rated sleep quality. This scale is widely utilized in sleep research and clinical practice to monitor alterations in sleep patterns and aid in the diagnosis and therapeutic assessment of sleep disorders.

#### Assessment of sleep architecture

Polysomnography (PSG) was conducted using the Tyco Sandman polysomnography respiratory monitoring system. PSG is a comprehensive sleep test that records various physiological parameters during sleep, including electroencephalography, electrooculography, electromyography, electrocardiography, airflow, respiratory effort, oxygen saturation, and leg movement. These measurements allow for the assessment of the patient's sleep architecture, degree of respiratory disturbance, and sleep quality, aiding in the evaluation of treatment effectiveness and patient's therapeutic needs. The AHI, total sleep time (TST), LSaO_2_, and longest apnea time (LAT) were compared preoperatively and 6 months postoperatively.

#### Assessment of cognitive function

The Montreal Cognitive Assessment (MoCA) (17) was used to evaluate the cognitive function of patients preoperatively and 6 months postoperatively. The MoCA, developed by Nasreddine et al. in 2005, is a highly sensitive, standardized tool for evaluating cognitive function, primarily used for screening mild cognitive impairment (MCI). The MoCA assesses seven cognitive domains, including executive functioning and visuospatial ability (5 points), naming (3 points), attention (6 points), language expression (3 points), abstraction (2 points), delayed recall (5 points), and orientation (6 points), with a total score of 30 points. A score of ≥26 is considered cognitively normal. To mitigate the influence of cultural factors, an additional point may be added to individuals with ≤12 years of education.

#### Assessment of quality of life

The Functional Outcomes of Sleep Questionnaire (FOSQ) (18) was used to assess the quality of life of patients preoperatively and 6 months postoperatively. The FOSQ is a self-rated questionnaire designed to assess the impact of sleep disorders, particularly sleep apnea, on an individual's daily functioning. It consists of 30 questions covering five domains: activity levels (physical capability), vigilance (attention and reaction speed), sexual relationships and intimacy (sexual satisfaction), productivity (ability to perform daily tasks), and social outcomes (social interactions and life satisfaction). Each item is scored on a scale from 1 to 20, with the total score reflecting the FOSQ score. A higher score indicates a lesser degree of impairment in quality of life due to sleep disorders.

All scales in this study were administered using standardized terminology by medically trained professionals, ensuring that participants fully understood the meaning of each option before completing the scales.

#### Statistical analysis

Data were analyzed using SPSS 23.0. Measurement data were expressed as mean ± standard deviation. Intergroup comparisons, specifically the differences in indicators between the control and study groups preoperatively and at 6 months postoperatively, were conducted using the independent sample *t*-test. For intragroup comparisons, the paired sample *t*-test was used to analyze changes within the same group of patients preoperatively and 6 months postoperatively. Categorical data were expressed as percentages and analyzed using the *χ*² test. Ranked data were evaluated using the rank-sum test. A value of *P* < 0.05 was considered statistically significant.

## Results

### Comparison of general data between the two groups

No statistically significant difference was observed between the groups in terms of sex, age, BMI, and course of disease (*P* = 0.824, 0.692, 0.430, 0.236). There was no significant difference in the baseline severity of OSAHS (mild/moderate/severe) between the two groups (*P* = 0.623), indicating good comparability of baseline condition (Table 1).

**Table 1:** Baseline characteristics of the two groups.

Variable	Control group (*n* = 54)	Study group (*n* = 45)	t/*χ*²/Z	*P*
Male/Female	30/24	26/19	0.049	0.824
Age (years)	52.3 ± 6.5	51.8 ± 5.9	0.397	0.692
BMI (kg/m²)	25.36 ± 2.02	25.68 ± 1.98	0.792	0.430
Disease duration (years)	12.32 ± 3.02	11.68 ± 2.15	1.192	0.236
Severity of condition (mild/moderate/severe)	10/26/18	6/23/16	0.492	0.623

Data are presented as mean ± standard deviation or number of cases. BMI: body mass index.

### Comparison of upper airway remodeling between the two groups

**Figure 3:**
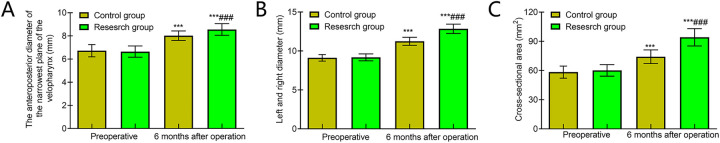
Effect of modified UPPP and pharyngeal and genioglossus exercises on upper airway remodeling in patients with OSAHS. **(A)**: Anteroposterior dimension, **(B)**: Transverse dimension, and **(C)**: Cross-sectional area of the narrowest plane of the palatopharynx. Compared with the preoperative period, ***P < 0.001; compared with the control group, ^###^*P* < 0.001. UPPP, uvulopalatopharyngoplasty; OSAHS, obstructive sleep apnea-hypopnea syndrome.

No statistically significant difference was observed in the anteroposterior, transverse, and cross-sectional areas of the narrowest part of the palatopharynx between the groups preoperatively (*P* = 0.355, 0.462, 0.138). In both groups, the anteroposterior, transverse, and cross-sectional areas of the narrowest part of the palatopharynx were significantly larger at 6 months postoperatively compared to preoperative measurements (*P* < 0.001 for all). The anteroposterior, transverse, and cross-sectional areas of the narrowest part of the palatopharynx were greater in the study group than in the control group at 6 months postoperatively (*P* < 0.001 for all) (Figure 3).

### Comparison of sleep quality between the two groups

Preoperatively, the SSS scores for the study and control groups were (5.16 ± 1.34) and (5.02 ± 1.15), respectively, and the intergroup comparison revealing no statistically significant difference (*P* = 0.058), indicating comparable baseline levels. At 6 months postoperatively, the SSS score in the study group was markedly lower than that in the control group (2.32 ± 0.72 vs. 2.64 ± 0.94), with a significant difference between groups (*P* < 0.01), suggesting that the combined treatment was more effective than conventional training in improving daytime sleepiness. Additionally, at 6 months postoperatively, the proportion of patients rating their sleep quality as “good” or “very good” in the study group was significantly higher than that in the control group (*P* = 0.025), suggesting that the combined training may be more effective than conventional training in improving sleep quality (Table 2).

**Table 2:** Comparison of self-rated sleep quality between the two groups.

Time	Group	Case	Very poor	Poor	Fair	Good	Very good	*Z*	*P*
Preoperatively	Control group	54	9 (16.67)	10 (18.52)	15 (27.78)	13 (24.07)	7 (12.96)	−0.08	0.936
	Study group	45	7 (15.56)	9 (20.00)	14 (31.11)	10 (22.22)	5 (11.11)		
At 6 months postoperatively	Control group	54	5 (9.26)	7 (12.96)	25 (46.30)	10 (18.52)	7 (12.96)	−2.20	0.028
	Study group	45	3 (6.67)	3 (6.67)	13 (28.89)	17 (37.78)	9 (20.00)		

Data are presented as *n* (%). χ² test was used for comparison between groups.

### Comparison of sleep architecture between the two groups

**Figure 4:**
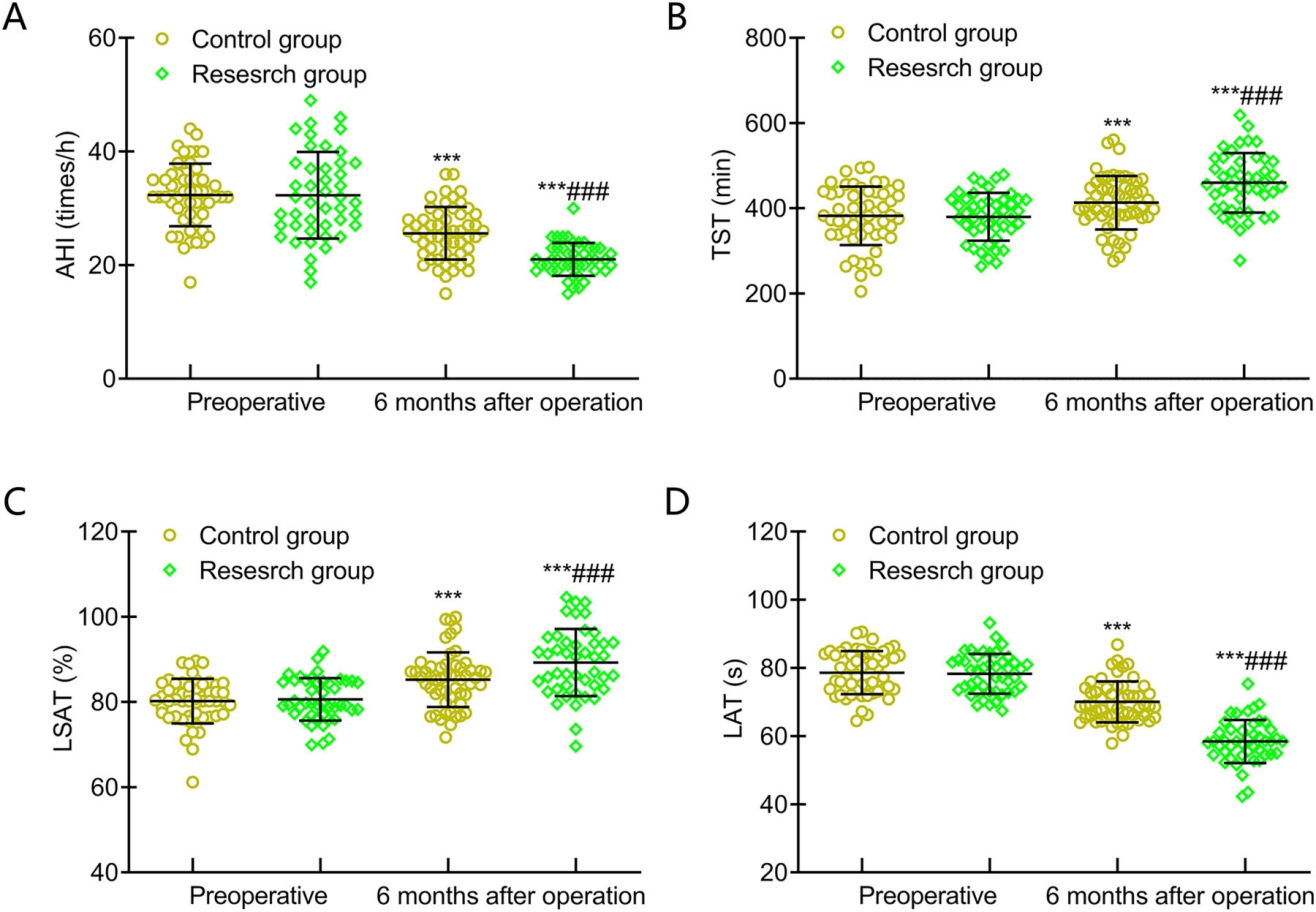
Effect of modified UPPP and pharyngeal and genioglossus exercises on sleep architecture in patients with OSAHS. **(A)**: AHI, **(B)**: TST, **(C)**: LSAT, **(D)**: LAT. Compared with the preoperative period, ***P < 0.001, compared with the control group, ^###^*P* < 0.001. AHI, Apnea Hypopnea Index; TST, total sleep time, LAT, longest apnea time; LSAT, lowest blood oxygen saturation; UPPP, uvulopalatopharyngoplasty; OSAHS, obstructive sleep apnea-hypopnea syndrome.

No statistically significant difference was observed in AHI score, TST, LSaO_2_, or LAT between the groups preoperatively (*P* = 0.971, 0.855, 0.707, 0.280). At 6 months postoperatively, both groups exhibited significantly decreased AHI score and LAT (*P* < 0.001 for all) and significantly elevated TST and LSaO_2_ compared with those assessed preoperatively (*P* < 0.001 for all). Additionally, the study group showed lower AHI score and LAT (*P* < 0.001 for all) and higher TST and LSaO_2_ than those of the control group at 6 months postoperatively (*P* = 0.001, 0.006) (Figure 4).

### Comparison of cognitive function and quality of life between the two groups

During the follow-up period, no serious postoperative complications or adverse events were observed in either group. No statistically significant difference was observed in the MoCA and FOSQ scores between the groups preoperatively (*P* = 0.313, 0.707). At 6 months postoperatively, both groups exhibited elevated MoCA and FOSQ scores compared with scores assessed preoperatively (*P* < 0.001 for all). Additionally, at 6 months postoperatively, the MoCA score in the study group was significantly higher than that in the control group (28.79 ± 2.46 vs. 27.01 ± 2.36; Cohen's d = 0.74, *P* < 0.001). The FOSQ score was also significantly higher in the study group compared with the control group (81.36 ± 10.58 vs. 64.41 ± 9.65; Cohen's d = 1.68, *P* < 0.001) (Table 3).

**Table 3:** Comparison of cognition function, quality of life, and respiratory pressure between the two groups (χ ± S).

Variable	Time	Control group (*n* = 54)	Study group (*n* = 45)	Mean difference	95% CI	Effect size (Cohen's d)	*P* value
MoCA score	Preoperatively	22.85 ± 3.23	23.43 ± 2.27	—	—	—	0.313
	At 6 months postoperatively	27.01 ± 2.36^***^	28.79 ± 2.46^***^	1.78	0.81–2.75	0.74	<0.001
FOSQ score	Preoperatively	53.65 ± 10.51	52.85 ± 10.49	—	—	—	0.707
	At 6 months postoperatively	64.41 ± 9.65^***^	81.36 ± 10.58^***^	16.95	12.88–21.02	1.68	<0.001
MIP (cmH2O)	Preoperatively	28.46 ± 5.68	27.34 ± 4.86	—	—	—	0.300
	At 6 months postoperatively	33.65 ± 4.84^***^	40.35 ± 5.02^***^	6.70	4.72–8.68	1.36	<0.001
MEP (cmH2O)	Preoperatively	37.16 ± 4.26	36.65 ± 3.38	—	—	—	0.517
	At 6 months postoperatively	42.15 ± 4.68^***^	51.84 ± 5.68^***^	9.69	7.59–11.79	1.88	<0.001

Data are presented as mean ± standard deviation. Compared with the preoperative period in the same group.

*** *P* < 0.001.

MoCA, Montreal Cognitive Assessment; FOSQ, Functional Outcomes of Sleep Questionnaire; MIP, maximal inspiratory pressure; MEP, maximal expiratory pressure.

### Comparison of respiratory pressure between the two groups

No statistically significant difference was observed in MIP and MEP between the groups preoperatively (*P* = 0.300, 0.517). At 6 months postoperatively, both groups exhibited significant increases in MIP and MEP compared to preoperative values (*P* < 0.001 for all). Additionally, the study group showed higher MIP and MEP than those of the control group at 6 months postoperatively (*P* < 0.001 for all) (Table 3).

## Discussion

This study demonstrated that the modified UPPP combined with pharyngeal and genioglossus exercises significantly enhanced sleep quality, cognitive function, and quality of life in patients with OSAHS. At 6 months postoperatively, the study group demonstrated significant improvements over the control group in terms of upper airway remodeling, respiratory pressure, sleep quality, sleep architecture, cognitive function, and quality of life. The results indicate that combination therapy not only effectively improves AHI and other physiological parameters, but also enhances patients' self-rated sleep quality. In the study group, a greater proportion of patients in the study group rated their sleep quality as “good” or “very good” compared to the control group, further corroborating the clinical benefits of the modified postoperative training regimen. Furthermore, the enhancement of sleep quality may positively influence cognitive function and quality of life. The findings of this study suggest that modified UPPP combined with pharyngeal and genioglossus exercises may be associated with improvements in multiple clinical indicators in patients with OSAHS.

Research has shown (19) that patients with OSAHS often experience impaired pulmonary function, which is closely associated with weakened upper airway muscle tone and airway collapse. The results of this study indicated that at 6 months after modified UPPP, the anteroposterior, transverse, and cross-sectional areas of the narrowest part of the palatopharynx, MIP, MEP, TST, and LSaO_2_ all increased, while AHI, and LAT decreased. Multiple studies (20, 21) found that modified UPPP significantly increased the cross-sectional area of the upper airway and reduced airway resistance, alleviating the anatomical burden on the upper airways. These findings are consistent with the results of the present study, indicating that modified UPPP improves sleep quality, sleep architecture, upper airway remodeling, and respiratory pressure in patients with OSAHS. Postoperative supplementary exercises targeting the pharyngeal and genioglossus muscles resulted in more pronounced improvements in patients. This effect may be attributed to the long-term exercises of these muscles, which enhances pulmonary functional residual capacity, improves pulmonary compliance, optimizes respiratory pressures, and facilitates upper airway remodeling. Simultaneously, this intervention regulates the muscle tone of the upper airway, reduces fatigue in the dilator muscles, and enhances pharyngeal wall compliance and upper airway activity during sleep (5). In recent years, an increasing number of studies have highlighted the importance of integrating surgical treatment with postoperative functional rehabilitation in patients with OSAHS. Previous research indicates that oropharyngeal training enhances upper airway muscle tone, decreases airway collapse, and improves clinical outcomes related to sleep (6). A recent study suggests that combining surgical intervention with functional training in a multimodal approach may further enhance postoperative respiratory stability and quality of life in patients with OSAHS (22). Therefore, the combination of modified UPPP and pharyngeal and genioglossus exercises may be an important factor contributing to improved clinical outcomes in patients with OSAHS.

Research indicates that patients with OSAHS, due to prolonged sleep deprivation and hypoxemia, frequently experience varying degrees of cognitive dysfunction and emotional disturbances (23, 24). If left untreated, these conditions may even lead to permanent brain damage (25, 26). This study revealed that the combination of modified UPPP with pharyngeal and genioglossus exercises effectively alleviated nocturnal hypoxemia, mitigated sleep structure disturbances, and improved negative mood, with superior outcomes compared to surgical intervention alone. It indicates that modified UPPP combined with postoperative pharyngeal and genioglossus exercises can further alleviate the impairment of cognitive function and reduce the negative emotions and psychological stress of patients.

However, this study has some limitations. First, it was a single-center retrospective analysis with a relatively small sample size, and patient grouping was based on the postoperative training actually received, which may introduce potential selection bias and limit causal inference. Second, the follow-up period in this study was limited to 6 months, which was insufficient to fully assess the long-term effects of the combined intervention on patients' cognitive function and quality of life. Furthermore, this study did not perform subgroup analyses based on OSAHS severity. Future research may consider stratified analyses to improve the precision of the findings. Additionally, this study did not include certain commonly used oxygenation indicators (e.g., LO2 and T90), which may lead to an incomplete assessment of hypoxic conditions. Moreover, multiple outcome measures were analyzed in this study without correction for multiple comparisons, which may increase the risk of type I error; therefore, the results should be interpreted with caution. Additionally, postoperative training adherence was not quantitatively assessed, and differences in adherence may serve as a potential confounding factor affecting the results. Finally, due to ethical and privacy constraints, complete intraoperative imaging was not available, and only schematic illustrations of the surgery were included, which may somewhat limit the comprehension of surgical details. Future large-scale, multicenter, prospective studies with extended follow-up, incorporating additional objective indicators and improved adherence assessment, are needed to further confirm the stability and reliability of these findings.

## Conclusion

In summary, the combination of the modified UPPP with pharyngeal and genioglossus exercises significantly improves sleep quality, cognitive function, and quality of life among 99 patients with OSAHS. Modified UPPP integrated with pharyngeal and genioglossus exercises yields more significant effects compared to conventional training.

## Data Availability

The original contributions presented in the study are included in the article/Supplementary Material, further inquiries can be directed to the corresponding author/s.
